# Spatiotemporal Trends and Distributions of Malaria Incidence in the Northwest Ethiopia

**DOI:** 10.1155/2022/6355481

**Published:** 2022-03-31

**Authors:** Teshager Zerihun Nigussie, Temesgen T. Zewotir, Essey Kebede Muluneh

**Affiliations:** ^1^Department of Statistics, College of Science, Bahir Dar University, Bahir Dar, Ethiopia; ^2^School of Mathematics, Statistics and Computer Science, College of Agriculture Engineering and Science, University of KwaZulu-Natal, Durban, South Africa; ^3^School of Public Health, College of Medicine and Health Sciences, Bahir Dar University, Bahir Dar, Ethiopia

## Abstract

Understanding and extracting noticeable patterns of malaria surveillance data at the district level are crucial for malaria prevention, control, and elimination progress. This study aimed to analyze spatiotemporal trends and nonparametric dynamics of malaria incidences in northwest Ethiopia, considering spatial and temporal correlations. The data were analyzed using count regression spatiotemporal models under the Bayesian setups, and parameters were estimated using integrated nested Laplace approximations (INLA). The region had a declining linear trend, and the average annual malaria incidence rate was 24.8 per 1,000 persons between 2012 and 2020. The malaria incidence rate was decreased by 0.984 (95% CI: 0.983, 0.986) per unit increase in months between July 2012 and June 2020. Districts found in the western and northwestern parts of the region had a steeper trend, while districts in the eastern and southern parts had a less steep trend than the average trend of the region. Compared to the regional level trend, the decreasing rate of malaria incidence trends was lower in most town administrations. The nonparametric dynamics showed that the monthly malaria incidence had a sinusoidal wave shape that varied throughout study periods. Malaria incidence had a decreasing linear trend changed across districts of the study region, and the steepness of trends of districts might not depend on incidences. Thus, an intervention and controlling mechanism that considers malaria incidences and district-specific differential trends would be indispensable to mitigate malaria transmission in the region.

## 1. Introduction

Malaria surveillance is a continuous, systematic collection, analysis, and interpretation of malaria data and is used as input for planning, implementation, and evaluation of public health practice [1, 2]. Understanding the trend of malaria surveillance data and extracting its noticeable patterns of prevalence across districts are crucial to inform concerned organizations about the state of malaria control and elimination [3].

Even though malaria morbidity and mortality have significantly declined in Ethiopia since 2001, 9% and 8% of the global *Plasmodium vivax* cases occurred in 2017 and 2018, respectively [4]. According to the world malaria report, the estimated number of malaria cases was 4,231,328 in 2020, significantly declining compared to 2010 [5]. Malaria is the major public health challenge in the Amhara region of Ethiopia [6]. The average malaria incidence rate was 23.51 per 1000 persons in the region between 2004 and 2014, and it was the fifth-largest incidence in the country [7]. The region has a decreased malaria burden, and the estimated prevalence was 4.6% in 2006, 0.6% in 2007, and 0.8% in 2011 [8]. More than 1.1 million cases occurred in 2012, with an annual incidence rate of 60 per 1000 persons, accounting for 19% of the national malaria cases. A cross-sectional survey in the 19 districts of more vulnerable revealed that malaria prevalence was 1.9% in 2013, and 40% of confirmed cases were asymptomatic [9].

Malaria transmission exhibits spatial and temporal variability due to diverse ecology, climate, altitude, topography, and human settlement patterns [10]. Even under lower transmission settings, districts have substantial malaria incidence variations in the region. Studies revealed that malaria risks have spatial, temporal, and spatiotemporal heterogeneity in the south and north Gondar zones [3, 6, 9]. Malaria incidence has spatial variations across districts of the region, influenced by trends or the number of malaria cases in their neighborhood districts [11]. Exploring and estimating malaria trends at the district level in considering the space-time interaction effect would reflect the entire picture of malaria transmission that would be crucial for planning, intervention, elimination, and control. Previous studies did not cover districts in the region, both with lower and higher malaria transmission scenarios. The regional level trend, differential trends of districts, and nonparametric dynamics of malaria incidence were not studied with the account of space-time interaction effects through spatial and temporal autocorrelations [6, 9]. In this study, malaria surveillance data from July 2012 and June 2020 were utilized to investigate and estimate global linear effects of time in a month, differential trends of districts, and nonparametric dynamics of malaria incidence.

## 2. Methods

### 2.1. Study Setting

**Figure 1:**
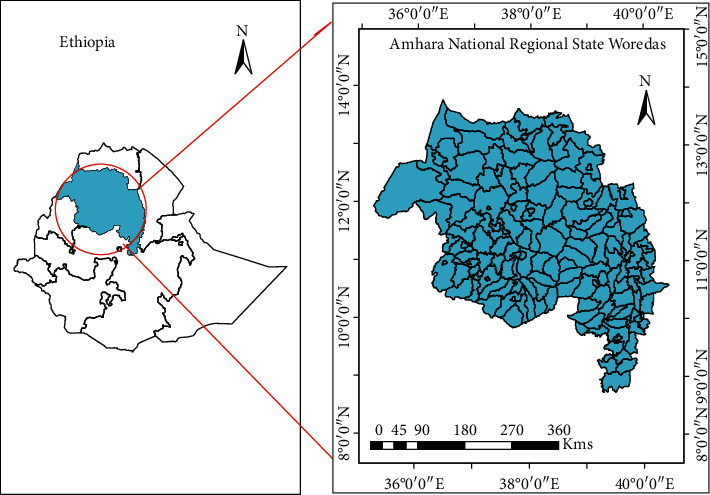
Study area map showing districts in the Amhara national regional state, Ethiopia in 2012.

This study was conducted in the Amhara National Regional State (ANRS), Ethiopia (Figure 1). The region is located in northwestern Ethiopia between 9°20′ and 14°20′ north latitude and 36°20′ and 40°20′ east longitude. The region has a monsoon climate, and its elevation ranges from 506 meters at the bottom of the Blue Nile gorge to 4,533 meters at Ras-Dajen mount [12]. The study area encompasses 152 rural and town districts that have had weekly malaria surveillance reports to the Amhara Public health institute (APHI) since 2012.

### 2.2. Study Design and Period

Using the weekly reported malaria surveillance data of Amhara Public Health Institute (APHI) from July 2012 to June 2020, a retrospective study design was used to investigate monthly spatiotemporal trends and space-time dynamics of malaria incidence.

### 2.3. Data

The weekly malaria surveillance data of rural districts, town administrations, general and specialized hospitals of the study region were obtained from Amhara public health institute (APHI), within the Public Health Emergency Management (PHEM) directorate. The weekly malaria data were reported using WHO EPI week that starts on Sunday and ends on Saturday.

The weekly malaria surveillance data encompass various information about districts, EPI-week, budget year, total malaria cases (clinical and confirmed), number of cases in different age groups, and types of *Plasmodium* species. This study used monthly malaria cases between July 2012 and June 2020, obtained by aggregating weekly surveillance data based on a month that had four or more days from a given WHO EPI-week. The estimated monthly population size of districts between 2012 and 2020, obtained from the central statistical agency of Ethiopia and Amhara National Regional State Planning Commission, is used as an offset in the model.

#### 2.3.1. Inclusion and Exclusion Criteria

The districts encompass various healthcare institutions such as health posts, health stations, and different levels of hospitals. Especially town administrations have general, referral, or specialized hospitals where patients get treatment with or without referral letters from the rural districts. Hence, malaria cases from the referral and specialized hospitals were not included in the analysis to preclude the overestimation of malaria incidence trends of town administrations.

### 2.4. Statistical Analysis

Spatiotemporal disease mapping models are widely used in disease surveillance studies [13] when the interest is identifying the underlying spatial and temporal patterns of diseases. Count spatiotemporal models were used to investigate and estimate malaria incidence trends at the district level in considering space-time interaction effects. Let *Y*_*it*_ be the number of malaria cases in the *i*^th^ district at time *t* and *n*_*it*_ be the size of the corresponding population at risk, for *i*=1,2,…,  *I*=152 and *t*=1,2,…,  *T*=96. Suppose *μ*_*it*_^*c*^ be the conditional expectations of the *Y*_*it*_ given random effects, then the disease mapping model is as follows:

(1) $$\begin{array}{c} \log\left(\mu_{it}^{c}\right)=\eta_{it}=\log\left(n_{it}\right)+\mu +u_{i}+v_{i}+T_{t}, \end{array}$$

where *μ* is the mean log-number of cases in overall areas, *u*_*i*_ and *v*_*i*_ are structured and unstructured spatial effects, respectively, and *T*_*t*_ represents temporal effects and can be specified in a parametric or nonparametric structure [14].

#### 2.4.1. Parametric Trend

The parametric time trend on the temporal components of the spatiotemporal model was introduced by Bernardinelli et al. [15], and the linear predictor given in equation (1) is written as follows:

(2) $$\begin{array}{c} \eta_{it}=\log\left(n_{it}\right)+\mu +u_{i}+v_{i}+\left(\beta +\delta_{i}\right)T_{t}, \end{array}$$

here, the equation includes the structured and unstructured spatial effects that represent between area-specific log-number of cases and overall mean rate, *β* is mean linear time trend, which represents global time effect, and *δ*_*i*_ is a differential trend (DT) that represents the difference between district-specific trends and regional level trends, which measures time and space interaction effects. For identifiability purposes, a sum-to-zero constraint is imposed on *δ*(*δ*_1_, *δ*_2_,…, *δ*_*n*_) and *u*(*u*_1_, *u*_2_,…, *u*_*n*_) and a value of *δ*_*i*_ < 0 implies that an area-specific trend is less steep than a mean trend, whilst a value of *δ*_*i*_ > 0 implies that the area-specific trend is steeper than the mean trend. The presence of a statistically significant differential trend in a given area is tested using posterior probability (PP), the Bayesian equivalent of the *p*-value [16]. A value of posterior probability, which is greater than 0.90, indicates some evidence of differential trend is greater than zero (greater than one in the exponential scales of parameters); that is, the area or district-specific trend is as steep as or steeper than the mean trend [15]. In disease mapping, spatial effects were considered random [15], and a Bayesian approach estimates parameters through assignments of prior distributions to the fixed parameters and spatial effect *u*_*i*_.

The prior distribution for the intercept is assumed to be zero-mean Gaussian with variance *σ*_*ρ*_^2^. The conditional autoregressive model proposed by Besag et al. [17] is used as a prior distribution for the spatially structured effects, that is,

(3) $$\begin{array}{c} \left[u_{i}|u_{j},i\neq j,\sigma^{2}\right]∼N\left(\mu_{i},\sigma_{i}^{2}\right), \end{array}$$

where *μ*_*i*_=(1/∑_*j*≠*i*_*w*_*ij*_)∑_*j*≠*i*_*w*_*ij*_*u*_*j*_, *σ*_*i*_^2^=*σ*^2^/∑_*j*≠*i*_*w*_*ij*_, and *w*_*ij*_=1, if **i** and *j*  are geographically adjacent districts and *w*_*ij*_=0 otherwise [18]. The uncorrelated spatial effect prior is defined as *v*_*i*_ ~ *N*(0,  *σ*_*v*_^2^) and is used to allow for uncorrelated extra variation. The differential trend, which accounts space-time interaction, is assumed to follow normal distributions with mean zero and variance *σ*_*δ*_^2^, that is, *δ*_*it*_ ~ *N*(0, *σ*_*δ*_^2^). In addition, noninformative prior distributions are considered for hyperparameters, so that the inference is based on the assumed model and observed data [19].

#### 2.4.2. Nonparametric Dynamic Trend

Bernardinelli et al. [15] evaluated spatiotemporal interactions on the disease risk by imposing a linearity constraint on differential temporal trends. The temporal trends in disease risk may be different for different spatial locations with a noticeable spatial variation. However, disease risk might not have linear temporal trends across areas. Knorr-Held [20] extended the linear time trend using a dynamic nonparametric formulation for the linear predictors underlying inseparable space-time effects. The log-link of the mean number of cases log(*λ*_*it*_)=*η*_*it*_ decomposes to

(4) $$\begin{array}{c} \eta_{it}=\log\left(n_{it}\right)+\mu +u_{i}+v_{i}+\alpha_{t}+\gamma_{t}+\delta_{it}, \end{array}$$

where *μ* is the overall malaria risk level, *u*_*i*_  and *v*_*i*_ represent unspecified features of district *i* that, respectively, do and do not display spatial structure. Similarly, *α*_*t*_ and *γ*_*t*_ represent the unspecified features of month *t* that, respectively, do and do not display temporal structure. The interaction between space and time effect is incorporated using *δ*_*it*_(*δ*_11_,…, *δ*_*nT*_), which would explain differences in the time trend of malaria cases for different areas or districts. The parameter *δ*_*it*_ is assumed to be Gaussian with precision matrix *λ*_*δ*_*K*_*δ*_, where *λ*_*δ*_ is unknown scalar and *K*_*δ*_=*I*_*v*_ ⊗ *I*_*γ*_=*I* is a prespecified structure matrix that is Kronecker's product of the structured matrices of the main effects, which are assumed to interact [21]. Here, the unobserved covariate effect at each pixel or area (*i*, *t*) assumed does not have any structure in space-time interactions, and all interaction parameters are prior independent [14, 20]. The spatial and interaction effects are assigned conditional autoregressive and normal prior distributions, respectively. In contrast, the structured temporal random effects are assigned random walk order 2 (*RW*2) prior distribution in order to account dynamic nature of the disease incidence. The random walk order 2 model is specified as follows: *α*_*t*_*|α*_*t*−1_, *α*_*t*−2_ ~ Normal(2*α*_*t*−1_+*α*_*t*−2_, *σ*^2^). The unstructured temporal effect (*v*_*i*_) has assigned a Gaussian exchangable prior distribution which is given as *v*_*i*_ ~ normal(0, *σ*_*v*_^2^=1/*τ*_*v*_). Moreover, hyperparameters are assigned noninformative flat priors.

#### 2.4.3. Parameter Estimation and Model Comparison

The Poisson spatiotemporal model is widely utilized for analyzing spatiotemporal count data. However, count data often display overdispersion, and using Poisson regression may underestimate the standard errors and overstate the significance of regression parameters [22]. In addition, a higher number of zeros in the data imposes problems, which might be occurred due to structural and sampling issues would be modeled using zero-inflated models [23, 24]. Districts located in the highlands areas, altitudes with 2500 and above meters, have a lower malaria transmission intensity, are malaria free, and might have zero monthly malaria cases, especially in the lower transmission seasons [9, 25–27]. The spatiotemporal models were compared using the deviance information criterion (DIC) and Watanabe–Akaike information criterion (WAIC). A spatiotemporal model with the smallest DIC value is used to fit and interpret results.

The model parameters and hyperparameters were estimated using Integrated Nested Laplace Approximations (INLA), which is a computationally efficient numerical approximation method for fitting complex spatiotemporal models and faster than Markov Chain Monte Carlo (MCMC) [28, 29].

## 3. Results

### 3.1. Temporal and Spatial Variation of Malaria Morbidity

**Figure 2:**
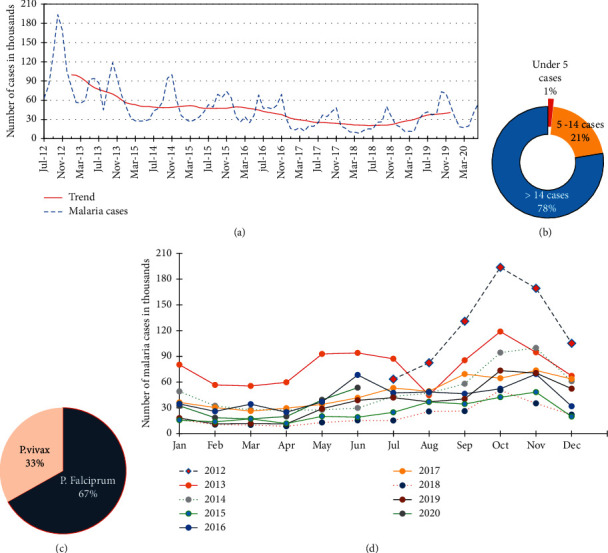
Total malaria cases by year, age group, months, plasmodium species, seasonal variation, and annual trends in the Amhara region, Ethiopia, between July 2012 and June 2020. (a) Monthly malaria cases and trends. (b) Total malaria cases by age groups. (c) Total confirmed cases by plasmodium species. (d) Monthly variations in the annual trends of malaria cases.

The malaria surveillance data showed that 4,565,506 malaria cases occurred in the Amhara region between July 2012 and June 2020, with a decline from July 2012 to June 2018 and an increasing trend since 2019. Monthly total malaria cases have fluctuated during months of the year, and there were higher variabilities at the beginning of the study periods between 2012 and 2015 (Figure 2(a)). The intensity of malaria infection varied among different age groups, and more than three-quarters (78%) of malaria patients were aged above 14 years. The remaining 1% and 21% of malaria patients were under 5 years and 5–14 years, respectively (Figure 2(b)). *Plasmodium falciparum* was a more prevalent malaria parasite in the Amhara region, accounting for 67% of the confirmed cases between 2012 and 2020 (Figure 2(c)).

The seasonal variation of yearly malaria cases is depicted in Figure 2(d), revealing that malaria cases were higher from September to November, following the primary rainy season between June and August, in 2012–2019. Monthly malaria cases were consistently higher in 2012 and 2013 than in other periods. The number of malaria cases reached the bottom level in 2018 and had a consistently lower number of patients throughout all months (Figure 2(d), red dot line). However, the number of malaria cases was higher in 2019 and 2020 compared to 2018, which has an increasing trend that might be due to various conditions limiting malaria prevention, control, and elimination endeavors of concerned bodies.

The average annual malaria incidence rate was 24.8 per 1,000 persons between 2012 and 2020 in the region. The annual malaria incidence rate was declined between 2012 and 2018, but there was a higher annual incidence rate in 2019. The result also indicates that total malaria and confirmed cases had consistently similar annual incidence rates between 2012 and 2020. The average annual incidence rate of malaria in patients and malaria in pregnant women were 9.68 and 16.93 per 100,000 persons per year, respectively, and reached the bottom level in 2018/19 (Table 1).

The annual malaria incidence rate was also varied on the types of *Plasmodium* species, and the result indicated that the average annual incidence rate of *P. falciparum* and *P. vivax* were 16.32 and 8.17 per 1,000 persons per year, respectively (Table 1). Besides, the result revealed that the annual malaria mortality rate was 2 per 1,000,000 persons per year. The annual incidence rates of total malaria and confirmed cases, *P. falciparum* cases, and malaria in pregnancy were lowest in the 2017/18 fiscal year. On the contrary, *P. vivax* cases and malaria in patients had the lowest incidence rate in 2018/19. However, the annual malaria mortality rate was stable throughout the study years (Table 1).

**Figure 3:**
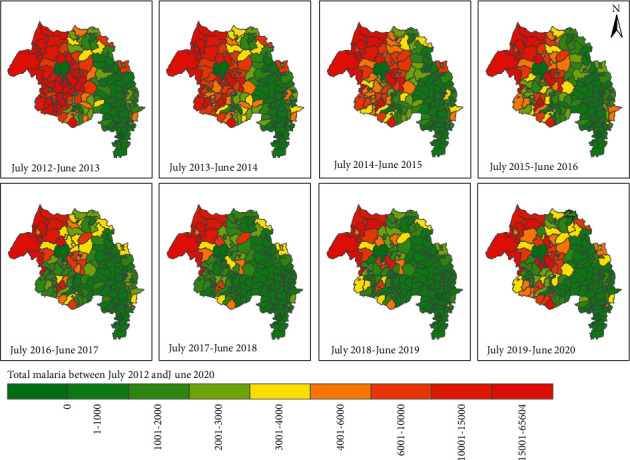
Spatial and temporal distribution of malaria incidences of districts in Amhara region, Ethiopia between 2012 and June 2020.

The annual malaria cases of districts in the study region are presented in Figure 3. Maps in Figure 3 depicted the spatial and temporal variations of malaria cases among districts of the Amhara region between 2012 and 2020 with a similar legend. The map showed that districts in the western Amhara region had higher malaria cases than districts located in the eastern parts of the region. Between 2012 and 2015, there were significantly higher malaria cases in North Gondar, South Gondar, Awi, East Gojjam, and West Gojjam zones.

Districts located in the highland areas of North Shewa, South and North Wollo, and Wag-Himra zones had fewer malaria cases either imported or infected through travel history (Figure 3). Further, the map showed that malaria incidence would have spatial and temporal autocorrelations due to the clustering of similar colors in the neighborhood districts throughout the study period. Hence, the spatiotemporal variation of malaria incidence would be considered to estimate and explore space-time trends and dynamics of malaria transmission in the region.

### 3.2. Parametric Spatiotemporal Trend

The model comparison result is presented in Table 2, and the spatiotemporal negative binomial model has the lowest DIC and WAIC values and is used to estimate spatiotemporal linear trends and nonparametric dynamics of malaria incidence.

The parametric spatiotemporal trends of malaria incidence results are presented in Table 3. The intercept and slope of time in a month were negative, indicating that the log scale of malaria incidence had decreasing linear trend between 2012 and 2020. The malaria incidence rate was reduced by a factor of 0.984 (95% credible interval: 0.983–0.986) for a unit increment in months in the study period. Moreover, the precision of spatial and space-time interaction effects was estimated, and their 95% credible intervals showed the presence of significant variations in spatial heterogeneity, spatial clustering, and differential trends across space over time.

**Figure 4:**
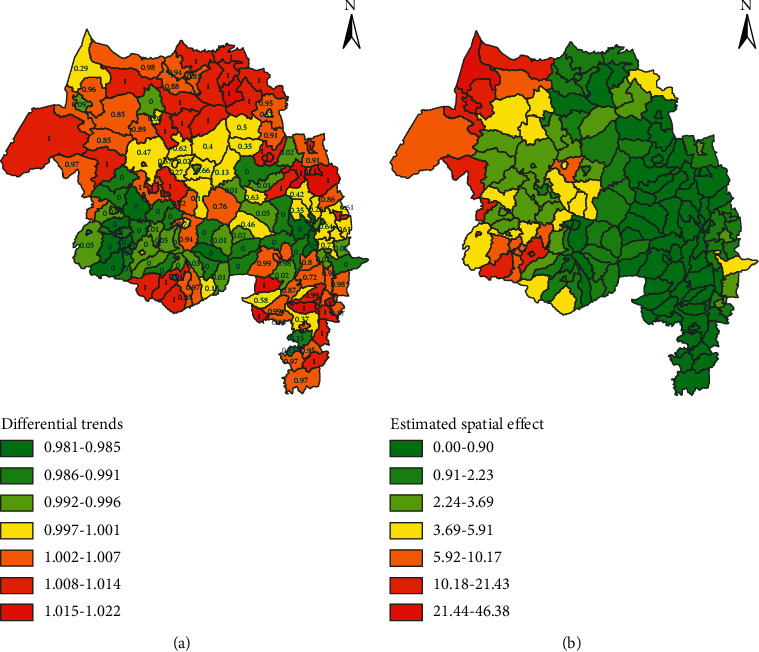
The estimated differential trends of districts with their corresponding posterior probabilities and spatial effects in northwest Ethiopia between 2012 and 2020. (a) Differential trends and the values within the map are posterior probabilities. (b) Estimated spatial effect for each district.

The space-time interaction effect is estimated using the differential trend representing the difference between district-specific trends and the average trend of the region and is depicted in Figure 4(a). The differential trends revealed that districts had a geographical variation on their district-specific trends. The values within Figure 4(a) are posterior probabilities used to test the presence of significantly different district-specific trends compared to the mean trend of the region. The 45 districts had a steeper trend than the mean trend since they had 0.9 or more posterior probabilities (Figure 4(a)). Figure 4(a) also depicted that most districts in the old North Gondar Zone, now reorganized into three zones named Central, West, and North Gondar, had a steeper trend than the average regional trend. Even though the North Shewa zone had a smaller number of malaria cases than other zones, most districts had a steeper trend than the mean trend of the region. On the contrary, districts located in West Gojjam and South Wollo Zone had a less steep trend than the average trend of the region (Figure 4(a)).

The study region has included 23 districts, which are solely town administrations, and most of them had a less steep malaria incidence trend than the average regional level trend. On the contrary, Bahir Dar, Lalibela, Kobo, Woldia, Bati, Debre Birhan, and Shewa Robit were town administrations that had a steeper trend than the mean trend of the region. Generally, districts shaded in yellow to light orange colors had a lower rate of decreasing trend than the average trend of the region (Figure 4(a)).

The spatial variation of malaria incidence was significantly explained using spatial effects through its components, that is, spatial clustering (structured) and spatial heterogeneity (unstructured). The 99.96% geographic variation of malaria incidence was explained using the spatial clustering of districts. The Moran's I statistic of the structured spatial effects was 0.443 (*P*-value = 0.0017 < 0.01), revealing the presence of significant spatial clustering of districts based on their malaria incidence. The estimated district/spatial effects and its map are given in Figure 4(b), indicating that as one goes towards the west and spatial effects of malaria incidence get higher. Malaria incidence had higher estimated spatial effects in the northwestern and western Amhara region, and it decreased as one moved towards the central and eastern parts of the region. Districts, mainly located in the highland fringe areas, had relatively smaller spatial effects, that is, in North Shewa, South Wollo, and North Wollo zones (Figure 4(b)).

### 3.3. Nonparametric Dynamic Trend of Malaria Incidence

The dynamic feature of the malaria incidence trend was estimated using negative binomial spatiotemporal dynamic model underlying separable space-time interaction effects, which has the smallest DIC value (Table 2). The nonparametric dynamic trends of malaria incidence were decomposed into structured and unstructured spatial and temporal effects, and their results are presented in Table 4. The result revealed that malaria incidence had significant spatial and temporal variation among districts of the study variations.

**Figure 5:**
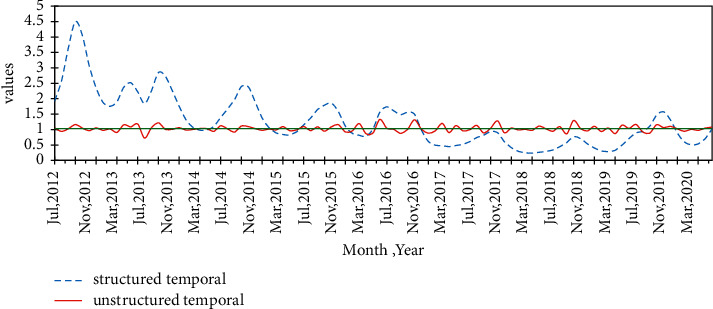
Posterior temporal trends of malaria incidence in the Amhara region, Ethiopia, between 2012 and 2020.

The structured and unstructured temporal dynamic trends of malaria incidence are shown in Figure 5. The structured temporal trend has an overall decreasing nonlinear trend between 2012 and 2018 and increased between 2018 and 2020 with a noticeable seasonal variation in each year. However, the unstructured temporal effects had random oscillations. The series plot had many peaks and changes over months that indicate the seasonality of malaria morbidity and higher temporal effects occurred between September and November throughout the study year.

## 4. Discussion

The focus of this study was estimating linear trends and dynamic features of malaria morbidity in the Amhara region using monthly surveillance data. More than 4.6 million people were confronted with malaria problems in the region between June 2012 and July 2020. The average malaria incidence rate was 24.8 per 1,000 persons per year between 2012 and 2020, which is a bit greater than in 2004–2014 [7]. The malaria incidence rate was declined three to fourfold in 2018 compared to 2012 and increased starting from mid-2018 might be due to drug and insecticide resistance, social, demographic, cultural, and behavioral beliefs and practices, and unreformed health infrastructure [30]. The result suggests that malaria incidence of the region followed a similar pattern of changes as the national and global malaria transmission patterns [4, 31]. Malaria is public health burden that affects individuals in all age intervals, and 78% of malaria patients were aged above 14 years. The most prevalent malaria parasite was *P. falciparum*, which accounted for 67% of confirmed cases in the region, a predominant malaria parasite in Ethiopia and the WHO African region [4, 6, 31–33]. The number of malaria cases was higher between September and December following the main rainy season in the region, and there were also considerably higher malaria cases from May to July. Seasonal variability might be attributed to the suitability of environmental conditions for the reproduction of mosquito vectors [34] and influenced by crop cycle, crop weeding, and grass cover of lands that appeared on and after rainy seasons [35].

The suitability of environmental conditions determines the distribution of *Plasmodium* species in space and time, and the spatiotemporal distributions of malaria have been related to this [36–38]. The annual malaria incidence varied across districts of the study region, and space-time distribution of malaria declined between 2012 and 2018. However, compared to 2018, most districts in the Amhara region had an increased number of malaria patients from 2019 to 2020. Districts in the northwestern and western parts of the region had higher annual malaria incidence than the eastern parts throughout the study period. Mainly, South Gondar, North Gondar, Awi, East Gojjam, and Wag-Himra zones had higher malaria incidence between 2012 and 2015, similar to a previous study finding in the west and near the border with Sudan and South Sudan [4]. The result is also supported by Alemu et al.'s [6] findings, which indicated that malaria transmission remained high in northwest Ethiopia between 2003 and 2012. On the contrary, districts located in the highlands of the North Shewa, North and South Wollo, and the Oromo special zone had lower malaria incidence between 2012 and 2020.

The estimated linear spatiotemporal trend suggested that the Amhara region had a decreasing malaria trend between 2012 and 2020; that is, between July 2012 and June 2020, per a unit increment in months of the year, the rate of malaria incidence decreased by a factor of 0.984, which is consistent with findings from previous studies conducted in Ethiopia between 2004 and 2016, 2011 and 2016, and 2013 and 2018 [3, 4, 32]. The result contradicted Taye et al.'s [7] predictions of an increased malaria trend in the Amhara region in 2015–2020, which might be occurred due to differences in data aggregation time scale and spatial units. Among the 152 districts, 45 had a significantly steep trend than the average trend of the region. The majority of districts with a higher malaria incidence in the North Gondar zone had a higher declining rate than the mean trend and had spatial disparity that is in line with the findings of Yalew et al. [9]. Among 23 town districts, about 70% of them had a less steep trend than the average regional trend, supported by Doumbe-Belisse et al.'s finding, revealed that malaria transmission has increased in most cities since 2003 [39]. However, Bahir Dar, Lal Yibela, Kobo, Woldia, Bati, Debre Birhan, and Shewa Robit were urban districts with a highly declining malaria incidence rate compared to the regional level trend.

The nonparametric dynamics of malaria incidence decomposed into spatial and temporal effects, each with structured and unstructured heterogeneity [20]. The structured monthly temporal dynamics fluctuated in the study periods and had seasonal variations, and higher incidence occurred following the main rain seasons between July and December [9]. Overall, malaria incidence decreased between 2012 and 2018 and began rising since 2019 across all months of the year. Compared to districts located in the eastern Amhara, the structured spatial effect of districts in western Amhara was higher. The results revealed that the North Gondar zone had a higher spatial effect due to peak malaria incidence. In contrast, North Shewa and South Wollo zones had lower spatial effects [6].

Spatiotemporal trend analysis is needed to provide insight and evidence supporting policy decision-making to prevent and control infectious diseases [40]. The parametric and nonparametric spatiotemporal trends are crucial for estimating area-level trends in considering spatial-time interaction effects. It is also used to detect areas that require emergency prevention and control interventions and can be reproduced anywhere with spatiotemporal areal data. The district-level trends would play a valuable role in evaluating the progress of malaria prevention and control performances of the districts' health offices. Furthermore, malaria prevention, control, and elimination could target districts with lower or higher malaria trends to achieve the malaria elimination target of the region. This study did not include climate, environmental, and other malaria transmission intervention impacts on the estimation of the nonparametric spatiotemporal dynamics of malaria, which is the limitation of the study.

## 5. Conclusions

The APHI malaria surveillance data have been used to explore and investigate the spatiotemporal linear and dynamic temporal trend of malaria incidence across districts of the Amhara region, Ethiopia. The Amhara region had a linearly declining malaria incidence trend between 2012 and 2020. The finding of this study revealed that districts in the western and southwestern parts of the Amhara region had a steeper trend than the average trend of the region. In contrast, districts in the eastern part of the region had a less steep trend than the average trend of the region. Further, two-thirds of urban districts had a less steep trend incidence than the average regional trend. Generally, monthly malaria incidence had sinusoidal wave dynamics that varied across months, and where there was an overall decreasing trend between 2012 and 2018. However, the trend of malaria incidence was reversed and showed an increasing trend. Thus, an intervention and controlling mechanism that considers malaria incidences and district-specific differential trends would be indispensable to mitigate malaria transmission in the region.

## Figures and Tables

**Table 1:** Annual malaria incidence for selected malaria indicators, Amhara region, Ethiopia.

Fiscal year	2012/13	2013/14	2014/15	2015/16	2016/17	2017/18	2018/19	2019/20	Average
Total malaria cases/1,000	54.64	30.34	26.92	25.61	16.44	11.73	12.46	20.28	24.80
Confirmed malaria cases/1,000	52.37	30.25	26.86	25.59	16.42	11.72	12.44	20.25	24.49
Malaria deaths/1,000,000	2.04	2.00	1.97	1.94	1.91	1.88	1.86	1.83	1.93
PF cases/1,000	32.10	19.04	17.61	16.95	11.00	8.60	9.61	15.68	16.32
PV cases/1,000	20.27	11.21	9.25	8.64	5.42	3.13	2.82	4.58	8.17
Malaria in-patients/100,000	32.99	16.65	8.53	6.37	3.59	2.98	2.45	3.84	9.68
Malaria in pregnancy/100,000	35.90	26.26	16.72	15.52	9.32	7.99	8.17	15.56	16.93

Source: The APHI malaria surveillance data, Amhara region, Ethiopia.

**Table 2:** Model comparisons of parametric and nonparametric spatiotemporal dynamics of malaria incidence.

Model	Distribution	Marginal log likelihood	DIC	WAIC
Parametric spatiotemporal trend	Poisson	−1,203,581.6	2,182,313.12	2,027,216.46
	Negative binomial	**−78,831.76**	**156,807.64**	**156,957.38**
	Zero-inflated negative binomial	−159,539.98	209,438.41	209,998.69

Nonparametric spatiotemporal dynamics	Poisson	−874,923.54	1,564,501.48	1,552,981.12
	Negative binomial	**−76,612.81**	**152,246.15**	**152,350.83**
	Zero-inflated negative binomial	−76,608.81	152,250.74	152,355.08

The bold values indicate the selected distribution for each model.

**Table 3:** Parametric spatiotemporal trends and fixed and precision parameters estimate malaria incidence in northwest Ethiopia between 2012 and 2020.

Variable	Mean	Mode	Standard deviation	0.025 quantile	0.975 quantile
Fixed effect					
Intercept	−6.494	−6.436	0.014	−6.522	−6.466
Time (month)	−0.016	−0.016	0.001	−0.018	−0.014

Precision of hyperparameters of the random effects					
Spatial heterogeneity	1830	372	2126.76	136.42	7230
Clustered spatial effect	0.237	0.237	0.029	0.182	0.297
Differential effect	11200	11100	1394.48	8628.56	14100

Dispersion parameter = 1.38 (test statistic = 2(ln*L*1 − ln*L*0) = 2,249,499.68; *p*-value <0.05).

**Table 4:** Nonparametric dynamic trend estimate of malaria incidence in northwest Ethiopia between 2012 and 2020.

Variable	Mean	Mode	Standard deviation	0.025 quantile	0.975 quantile
Fixed effect					
Intercept	−7.356	−7.356	0.039	−7.434	−7.278

Precision of hyperparameters random effects					
BYM model	0.251	3.254	0.038	0.177	0.327
RW2 model	13.48	11.32	3.84	8.08	22.96
Spatial heterogeneity	4.93	3.38	2.35	2.26	11.12
Temporal variability	34.21	29.57	9.40	20.43	56.99

^*∗*^BYM, Besag–York–Mollié; RW2, random walk order 2. Dispersion parameter = 1.36 (test statistic =  2(ln  *L*1 − ln  *L*0) = 1,596,619.48 (*p*-value <0.05).

## Data Availability

The malaria data used to support the findings of the study are available from the corresponding author upon request.

## Abbreviations

WHO: World health organization
EPHI: Ethiopian public health institute
APHI: Amhara public health institute
FMOH: Federal ministry of health
DIC: Deviance information criterion
WAIC: Watanabe–Akaike information criterion
PHEM: Public health emergency management
RDT: Rapid diagnostic test
EPI: Epidemiology
PF: Plasmodium falciparum
PV: Plasmodium vivax.
